# Child Social and Emotional Adjustment to First Grade: The Role of Emotion-Focused Parenting

**DOI:** 10.3390/bs15070855

**Published:** 2025-06-24

**Authors:** Shirley Agami-Turjeman, Roi Estlein

**Affiliations:** School of Social Work, University of Haifa, Haifa 3498838, Israel; sagamitu@campus.haifa.ac.il

**Keywords:** transition to first grade, child social adjustment, child emotional adjustment, parenting styles

## Abstract

The present study examined the associations between emotion-related parenting styles and children’s social and emotional adjustment to first grade. Hierarchical multiple regression analyses of data from 250 parents of children who had entered first grade showed that disapproving, dismissing, and laissez-faire parenting styles negatively predicted social adjustment, above and beyond other factors, such as the child’s emotion regulation and sociodemographic characteristics. Disapproving parenting also negatively predicted emotional adjustment, although this association was moderated by emotion regulation. Emotion-coaching parenting showed no significant associations with either social or emotional adjustment, indicating that scholars and professionals should pay particular attention to parenting that negates and rejects emotional aspects and is low in empathy and/or guidance. The current findings highlight the importance of considering emotional aspects of parenting for children’s adjustment to school, and they contribute to the understanding of how parental responses to children’s emotions play a role in their development and abilities to adjust during critical transition periods.

## 1. Introduction

Throughout their lives, all individuals experience inevitable changes. According to developmental theories, whereas many changes are not necessarily predicted (e.g., accidents, divorce, immigration), life transitions (e.g., leaving home as a young adult, the transition to parenthood, the transition to the empty-nest phase), which are culturally dependent and socially normative, are expected (Moos & Schaefer, 1986). During transitions, which are ongoing in nature (Estlein et al., 2022), individuals must adopt behavioral and emotional patterns in order to adjust to new demands, expectations, routines, and rules in the new environment (Estlein et al., 2024). These transitional periods often present various challenges as the individual navigates between different roles, assumes increased responsibility across various domains, and establishes new social networks (Taylor et al., 2014). One significant transition occurs in childhood during the critical period when children advance from the familiar and nurturing kindergarten setting to the more structured academic environment of first grade in school (Entwisle & Alexander, 1998; Marcineková et al., 2020).

### 1.1. The Transition to First Grade

Although the transition to formal schooling is universal and a normative change (Harper, 2015), it represents a complex and multifaceted process that demands simultaneous adjustments across emotional, social, and cognitive domains as children encounter more structured academic expectations, new behavioral norms, and different daily routines (Shoshani & Aviv, 2012). To contextualize this transition, it is important to consider the structure of the Israeli educational system. In Israel, children begin compulsory schooling in first grade at the age of six, following a year of mandatory, state-funded preschool education provided to all five year olds. While many children attend earlier preschool programs as well, the transition to first grade marks a distinct developmental milestone, as it entails a move from a play-based, nurturing preschool environment to a formal, academically oriented school setting characterized by increased cognitive demands, structured routines, longer school hours, and heightened expectations for behavioral regulation and independent functioning. These systemic characteristics underscore the emotional and social challenges that children may face during this transition. In this stage, children encounter structured, teacher-directed learning, emphasizing foundational literacy and numeracy skills (La Paro et al., 2006; Sink et al., 2007). Their success is formally assessed, and early academic performance is crucial due to the cumulative nature of the curriculum. Research consistently suggests that, without establishing strong learning and academic foundations in first grade, children may struggle to meet academic expectations in later years (Graziano et al., 2007; Murray & Harrison, 2011; Raver, 2003).

The shift to a more rigorous, structured environment requires students to adjust relatively quickly to new learning demands and instructional methods (Bossaert et al., 2011). For the first time, children confront the demands of a full academic day, requiring sustained attention to topics that they are not interested in, while simultaneously navigating physical constraints such as uncomfortable seating arrangements and expectations for maintaining appropriate behavior with minimal movement (Sink et al., 2007; Wong, 2003). Moreover, the first grade teacher’s educational practices significantly influence the learning environment and atmosphere (Sink et al., 2007)—another element that children must adjust to, including the nature of classroom tasks and homework assignments, which are entirely new experiences. These academic and environmental adjustments occur simultaneously with the need to develop enhanced emotional and social competencies, which are crucial for their positive adjustment to first grade. Importantly, emotional well-being and academic performance are closely intertwined, with each exerting a reciprocal influence during early schooling. Emotional adjustment facilitates learning, while academic challenges can, in turn, affect children’s psychological functioning (Graziano et al., 2007; McKown et al., 2016).

### 1.2. Socioemotional Adjustment to First Grade

Social competencies involve cultivating meaningful relationships with peers and educators through fundamental interaction skills, such as joining group play activities, initiating and sustaining age-appropriate conversations, demonstrating cooperation, practicing active listening, and respecting turn-taking protocols (Shoshani & Aviv, 2012). Complementarily, emotional competencies include developing healthy self-confidence, effectively regulating emotions across various classroom contexts, and successfully adjusting to the structured expectations and established norms within the school environment (Ladd et al., 1999). While school adjustment encompasses emotional, social, and academic domains, the current study focused specifically on the social and emotional aspects of adjustment, which, although developmentally salient at the start of formal schooling and closely aligned with parenting-related influences, have received significantly less attention in the literature. Academic adjustment was intentionally excluded from the scope of analysis to allow for a more in-depth examination of the affective and interpersonal dimensions of the first grade transition.

Social–emotional competencies are closely related to school adjustment in general and academic achievement in particular, as schools serve both as social environments and educational institutions (Zins et al., 2004). Consequently, well-developed social–emotional competencies are associated with interpersonal support from peers and teachers, which contributes to positive academic outcomes (McKown et al., 2016). The ability to socially and emotionally adjust to school has significant implications for developmental trajectories, with research showing that children who demonstrate strong adaptive competencies are more likely to achieve academic success and less likely to drop out in later years. In contrast, children who struggle with adjustment tend to experience greater difficulties both academically and socially (Ladd & Burgess, 2001).

The significant contribution of social and emotional competencies to a child’s elementary school adjustment necessitates a thorough examination of these developmental processes. Alongside the well-established research on academic adjustment, scholars have increasingly directed attention toward social and emotional competencies, recognizing their pivotal role in facilitating successful development, predicting psychological well-being across timeframes, promoting mental health, enhancing academic growth, and fostering overall educational success (Denham et al., 2003, 2014; Demirtaş-Zorbaz & Ergene, 2019). These capabilities specifically enhance first grade adjustment (Correia & Marques-Pinto, 2016) while serving as protective moderators between risk factors and critical developmental outcomes (Domitrovich et al., 2017).

### 1.3. The Environment and Social and Emotional Adjustment

Children’s development unfolds through ongoing environmental interactions, making it imperative to investigate the ecological conditions and processes that cultivate socioemotional competencies supporting smooth transitions to formal education. Bronfenbrenner’s (2005) bioecological theory provides an essential framework for understanding these environmental influences, conceptualizing development as occurring within a number of interconnected systems. Within this framework, the family microsystem emerges as the most immediate and influential context shaping early development (Shoshani & Aviv, 2012), playing a crucial role in nurturing fundamental competencies before school entry and profoundly influencing subsequent school adjustment. In particular, the parent–child relationship constitutes the primary and most influential connection from birth onward (Estlein, 2021).

Specifically, research on first grade transitions demonstrates that parental characteristics are associated with a child’s ability to adjust. For example, sociodemographic characteristics, such as higher parental education and greater economic stability, seem to enhance parental efficacy in fostering essential foundational skills in the child, which facilitates smooth integration into formal educational environments (Ben Shlomo & Taubman-Ben-Ari, 2017). Regarding specific parent–child interactions, maternal sensitivity during collaborative play strongly predicts children’s academic competencies in both preschool and first grade (Pianta & Harbers, 1996). Early family relationships—particularly the parenting quality, maternal education, sensitive caregiving, and linguistic stimulation through parent–child interactions—exert a more profound influence on school readiness and adjustment than any other childhood environmental factor (Shoshani & Aviv, 2012). Thus, the qualitative characteristics of early parent–child relationships serve as central determinants of children’s emotional and social adjustment capabilities during this critical developmental transition. The ways in which parents interact with their first-grade child—namely, their parenting styles—require a closer examination in order to understand their roles in the process of children’s adjustment to first grade.

### 1.4. Parenting Styles: An Emotion-Focused Model

Nearly a century of research has sought to understand parental influences on children’s behavioral, emotional, and social trajectories. Scholars have employed diverse parenting style typologies (e.g., Baumrind, 1967; Gottman et al., 1996) within broader investigations of parent–child socialization processes (Yafeh, 2016) to examine these developmental influences. A parenting style represents “a constellation of attitudes toward the child that are communicated to the child and create an emotional climate in which the parent’s behaviors are expressed” (Darling & Steinberg, 1993, p. 488). This multidimensional construct encompasses both deliberate parenting practices and nuanced, non-goal-directed interactions—including vocal tone, body language, attentional patterns, and emotional expressions—that collectively establish the affective context for child development. Baumrind (1967, 1971, 1991) pioneered a theoretical model organizing parenting styles into a clear taxonomy that remains foundational in contemporary research (Estlein, 2016). Based on varying combinations (high/low) of the quantity and nature of responsiveness and demandingness as two underlying dimensions, four parenting styles emerge (Baumrind, 1991; Maccoby & Martin, 1983): authoritative, authoritarian, indulgent/permissive, and rejecting/neglecting. Numerous studies have examined the associations between parenting styles and developmental outcomes in children and adolescents, indicating that children develop behavioral patterns that reflect the parenting style that they have experienced (Wolfradt et al., 2003).

Whereas Baumrind’s (1967, 1991) model refers to responsiveness as including both behavioral and emotional components expressed through parental warmth and general sensitivity to the child’s needs, it refers to general parenting attitudes and situations and may overlook parental responses to children’s emotions in emotionally salient interactions (Eisenberg et al., 1998). To address this gap, Gottman et al. (1996, 1997) have offered a dynamic and situationally grounded perspective, emphasizing parents’ real-time responses to children’s negative emotions, such as anger, fear, or sadness. In their emotion-focused parenting styles model, they highlight empathy, validation, and active guidance as central mechanisms of emotional socialization. The distinction between Baumrind’s and Gottman’s models reflects a conceptual shift from the general emotional climate to specific, emotion-related parenting practices embedded in everyday interactions (Hakim-Larson et al., 2006).

### 1.5. Gottman’s Emotion-Focused Parenting Style Model

Gottman’s theoretical framework is structured around two foundational dimensions: empathy and guidance. These complementary dimensions manifest through distinctive interpersonal processes that adults strategically deploy during emotionally salient exchanges with children (Rose et al., 2015). The empathy dimension involves recognizing, labeling, and validating a child’s emotional states regardless of behavior, creating a secure context that promotes self-awareness and facilitates constructive problem solving. The guidance dimension encompasses collaborative engagement with the child, which may include setting behavioral boundaries while emphasizing the joint exploration of alternative strategies to enhance emotional self-regulation and prevent future difficulties (Rose et al., 2015). Based on the integration of practices reflecting different levels (i.e., high/low) of empathy and guidance, Gottman’s model delineates four distinct parenting styles:

(1) Dismissing parenting, characterized by low empathy—where the adult perceives the child’s negative emotions as inconsequential and desires their rapid dissipation—and low guidance—where the parent abstains from collaborative problem solving and maintains that temporal progression will autonomously resolve difficulties. The dismissing parent employs strategies of ignoring or punishment, minimizes the significance of events that precipitate the child’s emotional responses, and interprets emotional expressions as manipulative tactics (Gottman et al., 1996, 1997). This style can be reflected in the following quote: “Stop crying already, it’s not that serious. You’ll be fine in a few minutes.”
(2) Disapproving parenting also combines low empathy and low guidance yet manifests more negatively and intensely than dismissing parenting. This style reflects low empathy, as the parent exhibits judgmental and critical attitudes toward the child’s emotional expressions, perceiving negative emotions as signs of weakness or defiance rather than legitimate experiences requiring validation and support. Emotional invalidation is central, as the parent discourages or reprimands expressions of sadness, fear, or frustration, fostering a sense that such emotions are unacceptable. It also demonstrates low guidance, as the parent does not teach adaptive emotional coping strategies but instead enforces the suppression or control of emotions through punitive measures, often equating emotional resilience with strict discipline. Children raised in this environment may struggle to articulate emotions and develop a sense of intrinsic defectiveness when experiencing negative feelings, contributing to emotional dysregulation (Havighurst et al., 2012). This style can be reflected in the following quote: “If you don’t stop this tantrum right now, it’s going to be very bad for you!”
(3) Laissez-faire parenting is characterized by high empathy, as the parent openly accepts and validates all emotional expressions without judgment. The child’s feelings are acknowledged and met with warmth, and there is no attempt to minimize, dismiss, or punish emotional displays. However, this style is marked by low guidance, as the parent fails to provide structure, problem-solving strategies, or behavioral boundaries, assuming that emotions should run their course naturally, without external intervention (Gottman et al., 1996, 1997). This style can be reflected in the following quote: “It’s okay to feel this way. Just let it all out in whatever way you need.”
(4) Emotion-coaching parenting is characterized by high empathy, as the parent demonstrates deep awareness and acceptance of both their own and their child’s emotional experiences. Emotion-coaching parents attentively observe their children’s emotional expressions, particularly sadness and anger (Schwartz et al., 2006), and respond to these emotions with understanding, warmth, and validation, allowing the child to feel heard and supported. Unlike laissez-faire parenting, this approach is also characterized by high guidance, as the parent actively teaches emotion regulation strategies, problem-solving skills, and socially appropriate ways of expressing emotions. Negative emotions are viewed as opportunities for connection and learning rather than as disruptions (Gottman et al., 1996, 1997). This style can be reflected in the following quote: “It’s okay to feel sad. Let’s take a deep breath together and think of a way to handle this.”

Research conducted among early childhood, elementary school-aged children, and adolescents demonstrates positive associations between emotion-coaching parenting and prosocial behavior and negative associations with behavioral problems (e.g., Katz & Hunter, 2007; Katz & Windecker-Nelson, 2004; Shipman et al., 2007). In a three-year longitudinal study, Katz and Gottman (1997) found that emotion coaching in parents of kindergarten-aged children functioned as a buffer against the adverse effects of marital distress on children’s behavior, as well as social problems, and was positively associated with academic achievement. Additionally, the research literature supports the contribution of positive parental responses to children’s emotions (e.g., acceptance and problem solving) to emotion regulation capabilities in school-aged children, whereas negative parental responses (e.g., emotional dismissal and criticism) contribute to regulatory difficulties and constitute a direct risk factor for increased behavioral problems (Eisenberg & Fabes, 1994; Lunkenheimer et al., 2007; McDowell et al., 2002).

### 1.6. The Current Study

Despite the widely acknowledged importance of family as the first social environment for children and a primary socialization agent (Walsh, 2003), significant gaps remain in our understanding of the specific mechanisms through which parent–child relationships can influence children’s adjustment capacities. While the family constitutes a factor of decisive importance (McGoldrick & Carter, 2003) in shaping a child’s emotional and social adjustment before school entry (Shoshani & Aviv, 2012), the literature has largely overlooked the emotional dimension of parenting in favor of behavioral approaches. Moreover, most studies examining parenting styles have relied on Baumrind’s (1967) model, which emphasizes behavioral aspects and general parental attitudes rather than emotional dimensions. This information has been extremely important; however, much less is known about how children develop skills and behavioral patterns that reflect their parents’ emotional socialization practices (Wolfradt et al., 2003). The relationship between parent–child interactions and children’s adjustment capacity across contexts is extensively documented (Martin et al., 2019), yet the emotional pathways of this influence remain underexplored.

The current research addresses this gap by adopting Gottman et al.’s (1996, 1997) emotion-focused parenting model, which explicitly considers parents’ perceptions and responses to emotions within the parent–child relationship. By examining how emotion-oriented parenting styles—particularly emotion-coaching versus emotion-dismissing approaches—contribute to children’s social–emotional skills, we can better understand the mechanisms facilitating successful adjustment during the critical transition to first grade.

This study proposes that parenting styles that directly address the emotions of both the parent and child (such as emotion coaching) will enhance the ability of children to socially and emotionally adjust (Ramsden & Hubbard, 2002), whereas practices that negate emotional aspects may promote a lower adjustment capacity. The importance of this relationship is especially pronounced during the transition to school, when children must deploy internalized emotional skills to navigate new social and academic demands. Formally stated, our research hypothesis is as follows:

**H1.**  *Emotion-focused parenting styles predict child social and emotional adjustment to first grade.*

Specifically, we propose the following:

**H1a.**  *A dismissing parenting style negatively predicts child social and emotional adjustment to first grade.*

**H1b.**  *A disapproving parenting style negatively predicts child social and emotional adjustment to first grade.*

**H1c.**  *A laissez-faire parenting style negatively predicts child social and emotional adjustment to first grade.*

**H1d.**  *An emotion-coaching parenting style positively predicts child social and emotional adjustment to first grade.*

## 2. Materials and Methods

### 2.1. Participants

The study’s sample consisted of 250 Israeli parents (221 mothers and 23 fathers; 6 did not report their sex) of a first-grade child (115 boys, 47.1%; 129 girls, 52.9%). The sample size was a priori determined by a power analysis using G*Power version 3.1.9.7 in order to reach 95% power to detect medium to small 0.06-sized effects (sample size was determined at 209). Data were collected approximately three months after the children had transitioned to first grade in order to reflect on the adjustment experience within a reasonable timeframe after starting school yet not immediately at the beginning of first grade. The mean age of the participating parents was 39.27 years (SD = 5.32). The majority of parents (83.6%) had completed higher education, with 44.1% holding a Bachelor’s degree and 36.4% holding a Master’s degree or higher. In terms of income, most participants (76%) reported an average or above-average gross income for a household with children in Israel, and the rest reported incomes that were significantly lower than average (3.2%), slightly below average (6%), and close to/almost similar to the average (14.8%). Nearly half of the participants (48.2%) identified as secular, 35.2% described themselves as culturally or moderately religious, and 16.6% identified as traditional or religious.

### 2.2. Procedure

Following approval from the University of Haifa’s Faculty of Social Welfare and Health Sciences’ Ethics Committee, invitations calling for participation in the study, containing comprehensive study information, informed consent documentation, and access to the online questionnaires, were distributed across diverse parent communities (including specialized Facebook groups, online forums, and the principal investigator’s professional network). Participants provided explicit informed consent prior to accessing the questionnaire battery, which was administered via the Qualtrics survey platform. The platform’s response validation features ensured complete data collection for all participants.

### 2.3. Measures

Parenting Style. To assess parents’ emotion-focused parenting, we employed the Emotion-Related Parenting Style Self-Test (ERPSST-L; Hakim-Larson et al., 2006). The original questionnaire, comprising 81 items rated as “true” or “false”, was developed by Gottman and DeClaire (1997) and is based on the meta-emotion philosophy parenting style interview (Katz & Gottman, 1997). Hakim-Larson et al.’s (2006) version presents a Likert scale format and assesses parental agreement (1—“always untrue”, 5—“always true”) with statements reflecting their attitudes and responses to anger and sadness in parenting contexts and characterizes the parent’s style accordingly. Scores were calculated by averaging responses on items representing a specific style and dividing them by the number of items in each scale. Higher scores in a particular style indicate greater utilization of practices associated with this style. In the current study, Cronbach’s alpha reliability tests were conducted for each parenting style. For the dismissing parenting style, Cronbach’s alpha reliability of α = 0.51 was obtained; for the disapproving parenting style, α = 0.62; for the laissez-faire parenting style, α = 0.43; and, for the emotion-coaching parenting style, α = 0.79.

Emotional and Social Adjustment to First Grade. To evaluate the child’s social and emotional adjustment to first grade, we used Smilansky and Sheftaya’s (2001) Scale for Assessing Adjustment in Kindergarten and School. This scale comprises eighteen questions and assesses adjustment across three domains: academic, social, and emotional. Each domain contains six items. For the purpose of this study, the six items referring to academic adjustment were excluded. Each question includes five statements describing a rating of the child’s behavior, requiring the selection of the best option according to the participating parent. For example, in the emotional adjustment domain, for “Is the child disciplined?”, ratings range from 1—“Always disrupts and resists routine and regular order” to 5—“Always disciplined and never needs to be punished.” In the social adjustment domain, for “Is the child sociable?”, ratings range from 1—“Mostly isolates themselves” to 5—“Easily forms many social connections and maintains them.” The emotional adjustment scale yielded Cronbach’s alpha reliability of α = 0.66, and the social adjustment scale yielded Cronbach’s alpha reliability of α = 0.57.

Control variables included the child’s gender and birth order (firstborn, middle child, youngest) and family income, as well as the parent’s education and level of religiosity. As previous studies indicate associations between a child’s emotion regulation and their adjustment to first grade (Harrington et al., 2020; Russell et al., 2016), we wished to control for emotion regulation, using Shields and Cicchetti’s (1997) Emotion Regulation Checklist (ERC), a 24-item parental report instrument to assess children’s emotion regulation. The score is calculated by reversing all negative items in the questionnaire and computing the average for all 24 items, so that a higher score indicates better regulation abilities. The emotion regulation scale yielded Cronbach’s alpha reliability of α = 0.65.

### 2.4. Data Analysis

Data analysis was conducted using SPSS 28.0. Preliminary analyses included the examination of descriptive statistics (frequencies, means, and standard deviations) and assessment of measurement properties for all variables. We continued the preliminary analyses by examining bivariate correlations among all variables in our study to document the nature of the associations between them. We then tested our research hypotheses using a series of hierarchical multiple regression models, in which predictors were entered in successive blocks to assess their incremental contributions to the explained variance in children’s adjustment. In addition to evaluating statistical significance, we assessed the standardized effect sizes of the regression coefficients to interpret the magnitude of the observed associations. Following established conventions in developmental psychology, we considered standardized beta coefficients (β) of approximately 0.10 as small, 0.30 as medium, and 0.50 or above as large effects (Cohen, 1988; Funder & Ozer, 2019). This approach allows for a more meaningful interpretation of findings beyond *p*-values alone. This analytical approach allowed for a systematic examination of whether controlling for sociodemographic variables and potential mediating/moderating factors would attenuate or eliminate the direct associations between the predictor variables and the adjustment outcomes. In terms of missing data, across all variables, a proportion of approximately 14% of the data was missing across the key study variables. Following Little et al. (2014), the MCAR test was non-significant, χ^2^(13) = 9.15, *p* = 0.761, indicating that data were missing completely at random. We employed full information maximum likelihood (FIML) estimation to use all the available information from the observed responses and maximize the accuracy and statistical power.

## 3. Results

Descriptive statistics are presented in Table 1. Table 1 also presents the bivariate correlations among all study variables, which revealed significant associations between three of the parenting styles and children’s social adjustment to first grade, namely dismissive parenting (r = −0.18, *p* < 0.05), disapproving parenting (r = −0.26, *p* < 0.01), and laissez-faire parenting (r = −0.17, *p* < 0.05). For emotional adjustment, only disapproving parenting demonstrated a significant negative association (r = −0.21, *p* < 0.01). Surprisingly, emotion coach parenting did not significantly correlate with either social or emotional adjustment (r = −0.01, *p* = n.s.; r = 0.06, *p* = n.s., respectively). Several control variables showed positive associations with child adjustment outcomes: child gender (r = 0.18, *p* < 0.05) and family income (r = 0.14, *p* < 0.05) with social adjustment, and child birth order (r = 0.15, *p* < 0.05) and family income (r = 0.15, *p* < 0.05) with emotional adjustment. Emotion regulation was significantly associated with both emotional adjustment (r = −0.55, *p* < 0.01) and social adjustment (r = −0.26, *p* < 0.01) in class.

Next, we implemented a step-by-step hierarchical regression model for each of our hypotheses to examine whether emotion-focused parenting styles predicted child social and emotional adjustment to first grade above and beyond the correlated controlling variables. Starting with H1a, our final model (*R*^2^ = 0.161, *F* = 9.00, *Cohen’s f*^2^ = 0.19, *p* < 0.001; see Table 2) indicated a significant negative association between dismissing parenting and social adjustment above and beyond other variables (*b* = −0.23, *p* < 0.01), reflecting a moderate effect size. In terms of the controlling variables, child gender emerged as a positive predictor (*b* = 0.16, *p* < 0.01), indicating that first-grade girls socially adjust better than first-grade boys; this association reflects a small effect. Emotion regulation displayed a substantial negative association with social adjustment (*b* = −0.42, *p* < 0.001), corresponding to a moderate-to-large effect. Recall that there was no significant association between the dismissing parenting style and emotional adjustment to first grade; therefore, no regression analysis was conducted.

In terms of H1b, our final model (*R*^2^ = 0.175, *F*(186) = 9.84, *Cohen’s f*^2^ = 0.21, *p* < 0.001; see Table 3) indicated a significant negative association between a disapproving parenting style and social adjustment, even after controlling for other variables (*b* = −0.20, *p* < 0.001), reflecting a moderate effect size. In terms of the controlling variables, child gender emerged as a positive predictor (*b* = 0.16, *p* < 0.01), indicating that first-grade girls socially adjust better than first-grade boys; this association represents a small effect. Emotion regulation displayed a substantial negative association with social adjustment (*b* = −0.34, *p* < 0.01), corresponding to a moderate effect size.

Additionally, our final model (*R*^2^ = 0.344, *F*(188) = 24.68, *Cohen’s f*^2^ = 0.52, *p* < 0.001; see Table 4) to test the association between disapproving parenting and emotional adjustment indicated a significant negative association between the variables across the first three steps (Step 1: *b* = −0.23, *p* < 0.01; Step 3: *b* = −0.21, *p* < 0.01), both reflecting moderate effect sizes, but this became non-significant in Step 4 (*b* = −0.11, *p* = n.s.), indicating a small and negligible effect size after introducing emotion regulation. These results suggest that emotion regulation moderates the association between disapproving parenting and emotional adjustment to first grade. As for the control variables, child gender positively predicted the outcome variable (*b* = 0.18, *p* < 0.01), representing a small effect, and emotion regulation displayed a substantial negative association with emotional adjustment (*b* = −1.06, *p* < 0.001), reflecting a large effect.

Continuing with H1c, our model (*R*^2^ = 0.136, *F*(187) = 9.82, *Cohen’s f*^2^ = 0.16 *p* < 0.001; see Table 5) indicated a significant negative association between laissez-faire parenting and social adjustment, even after controlling for other variables (*b* = −0.16, *p* < 0.05), reflecting a small-to-medium effect. In terms of the controlling variables, child gender emerged as a positive predictor (*b* = 0.16, *p* < 0.01), reflecting a small effect, and emotion regulation displayed a negative association with social adjustment (*b* = −0.43, *p* < 0.001), reflecting a moderate-to-large effect. The excluded variable analysis indicated that family income was not included in the model, as it did not significantly contribute to predicting social adjustment when controlling for the other variables. Recall that there was no significant association between the laissez-faire parenting style and emotional adjustment to first grade.

Finally, the analysis for the testing of H1d revealed no significant associations between emotion-coaching parenting and children’s social or emotional adjustment to first grade. Given that the bivariate correlations between emotion coaching and the two adjustment variables were weak and non-significant (r = −0.01 and r = 0.06, respectively), we followed standard statistical recommendations (e.g., Aiken & West, 1991) and did not include this predictor in the regression models. Including variables with negligible zero-order associations may reduce the model interpretability and statistical power.

## 4. Discussion

Like other life transitions, the transition to first grade can be—and usually is—stressful for children, due to the demands to adjust to numerous changes and challenges. With the parent–child relationship constituting the primary and probably the most influential relationship in the child’s environment, the ways in which parents exert their parenting are associated with their child’s ability to adjust (Ben Shlomo & Taubman-Ben-Ari, 2017). The significant role that parenting plays in children’s short- and long-term psychological adjustment has been a central principle in developmental and family psychology (Estlein, 2021). Research concerning the potential influences of parents on children’s development has mainly focused on parental discipline-centered behavior and attitudes toward authority and control (e.g., Baumrind, 1967, 1971; Maccoby & Martin, 1983). Limited attention, however, has been devoted to examining the emotional aspect of parenting and to parents’ perceptions of and responses to their own and their children’s feelings. The purpose of the current study was to address this gap in the literature by examining the associations between emotion-focused parenting styles and first-grade children’s social and emotional adjustment to school. Our findings supported some of our research hypotheses, documenting negative associations between the three emotion-negating styles (i.e., dismissing, disapproving, and laissez-faire) and child’s adjustment to first grade, but they indicated no significant associations between the emotion-coaching style and a child’s social and emotional adjustment. In what follows, we discuss our findings in the context of the parenting style and developmental psychology literature and consider the implications that our results have for research on child adjustment, the transition to first grade, and parent–child relationships.

### 4.1. Dismissing Parenting Style

The first research hypothesis (H1a) predicted a negative association between the dismissing parenting style and child social and emotional adjustment to first grade. The findings partially supported our hypothesis, indicating a significant association between dismissing parenting and social adjustment, but not with emotional adjustment. These results align with previous findings that suggested that children whose parents are characterized by a dismissing parenting style are low in social skills, particularly compared to children whose parents rely on an emotion-coaching parenting style (Denham et al., 1997; Gottman et al., 1996), and they add to our knowledge by highlighting the specific potential impact of such parenting on children’s ability to adapt during the critical transition to formal schooling.

Specifically, in first grade, where social skills are important because they facilitate peer acceptance and academic collaboration, the role of parents’ parenting styles becomes particularly pronounced (Pianta & Walsh, 1996). As children are required to function independently within a new and demanding social environment, often for the first time, this setting places increased importance on prior socialization experiences within the family, making parenting practices especially consequential. Moreover, it may be that a dismissing parenting style, which is characterized by low empathy and low guidance and minimizes the significance of events that generate emotional responses, constitutes a model for the child in developing social relationships with their classmates. In this sense, through a social learning modeling process (Bandura, 1977), the child may present similar dismissing behavior in class, which makes it difficult for him/her to establish proper positive social relationships with their peers.

Interestingly, unlike social adjustment, the association between dismissing parenting and emotional adjustment was not significant. We believe that this distinction may point to the influence of additional individual and environmental factors in shaping emotional adjustment during school transitions, such as child temperament, teacher–child relationships, or the classroom climate (Rimm-Kaufman & Pianta, 2000). Recent research supports the view that emotional adjustment is associated with a complex interaction among dispositional and contextual factors, which may attenuate the effects of parenting behaviors that are more passive in nature (Lunkenheimer et al., 2007; Zarra-Nezhad et al., 2015). Furthermore, this finding may suggest that emotional adjustment is a more internally regulated process, less immediately reactive to parental modeling compared to observable social behaviors, which may be more easily shaped by early relational experiences.

### 4.2. Disapproving Parenting Style

Our next hypothesis (H1b), predicting a negative effect of disapproving parenting on children’s social and emotional adjustment to first grade, was fully supported. Notably, whereas the association between the disapproving parenting style and social adjustment remained significant above and beyond other variables, the association with emotional adjustment became non-significant when emotion regulation was added to the model, suggesting that the effect of disapproving parenting on emotional adjustment is moderated by the child’s emotion regulation abilities. Recall that disapproving parents set rigid boundaries for their children’s emotional expressions, believe that negative emotions are a sign of weakness, and, therefore, employ harsh emotion-disapproving tactics (Gottman & DeClaire, 1997). The numerous novel demands and changes in transition periods in general, and in the transition to first grade in particular, often generate feelings of stress (Compas et al., 1986) and increased psychological distress (Chung et al., 1998). This experience can invoke increased negative emotions and expressions from the child, which, in the eyes of the disapproving parent, are perceived as invalid and prohibited. By prohibiting and negating such feelings and behaviors, disapproving parents cause the child experiencing these emotions to feel that they are inappropriate and, thus, suppress them. Suppressing strong feelings can increase frustration and stress (Brown & Taghehchian, 2016), which can manifest in aggressive behavior (Dollard et al., 2013) toward peers (Kang et al., 2019), sabotaging social relationships with classmates.

Emotionally, however, this process may be moderated when children can rely on effective emotion regulation skills. Our results, indicating that the association between disapproving parenting and emotional adjustment to first grade is moderated by child emotion regulation, suggest that, when children find ways to soothe and reassure themselves, they may more effectively adjust to the class environment (McDowell et al., 2002). These findings are consistent with previous findings that maintained that negative parental reactions (e.g., criticism) constitute a direct risk factor contributing to more behavioral problems (Eisenberg & Fabes, 1994; Lunkenheimer et al., 2007; McDowell et al., 2002). Furthermore, longitudinal analyses indicate that negative parental reactions to children’s emotions are associated with emotional difficulties (e.g., in emotion regulation; Eisenberg et al., 1998, 1999). The current study adds a unique focus on the role of disapproving parenting styles in the adjustment to first grade, indicating that, during significant transition periods, emotional aspects—particularly the disapproval of such aspects—in the parent–child relationship are central in the adjustment process.

### 4.3. Laissez-Faire Parenting Style

Next, H1c predicted that the laissez-faire parenting style would negatively predict child social and emotional adjustment to first grade. Similarly to dismissing parenting, the laissez-faire parenting style demonstrated a significant negative association with social adjustment, even after controlling for other variables, but not with emotional adjustment. This pattern may reflect the shared feature of both parenting styles: a lack of structured emotional guidance and regulation support. Laissez-faire parents tend to accept their children’s emotions but provide minimal boundaries, structure, or strategies for managing them (Gottman et al., 1996). As a result, children may enter the school environment without clear expectations regarding appropriate emotional expression or social behavior. In the context of first grade—where children are expected to navigate peer dynamics, follow classroom rules, and collaborate with others—this absence of behavioral guidance may be particularly detrimental to social adjustment. Social functioning often relies on external modeling and reinforcement, which laissez-faire parenting may fail to provide (Zarra-Nezhad et al., 2015). Through limited feedback and inconsistent expectations, this parenting style may leave children socially unprepared for structured interactions with peers and teachers.

In contrast, emotional adjustment may depend more heavily on individual traits (such as temperament) and the availability of external emotional supports in the classroom context (Lunkenheimer et al., 2007; Rimm-Kaufman & Pianta, 2000). Thus, while the laissez-faire style may hinder the development of socially adaptive behaviors, its association with emotional adjustment may be weaker or more context-dependent.

### 4.4. Emotion-Coaching Parenting Style

Our final hypothesis (H1d) predicted that an emotion-coaching parenting style—reflecting high parental empathy and high parental guidance—would positively predict children’s social and emotional adjustment to first grade. Contrary to our expectations, our results showed no significant associations between emotion-coaching parenting and social and emotional adjustment.

These results may be somewhat surprising, as some studies have documented associations between the emotion-coaching parenting style and children’s positive developmental outcomes (e.g., Gottman et al., 1996; Katz & Hunter, 2007; Katz & Gottman, 1997; Katz & Windecker-Nelson, 2004; Shipman et al., 2007). However, the literature has not always been consistent, with studies also reporting no significant associations between this parenting style and developmental outcomes (e.g., Lagacé-Séguin & Coplan, 2005; Lunkenheimer et al., 2007). The findings of the current study seem to join the latter line of studies.

This inconsistency can be explained in at least three ways. First, although related, different outcome variables were examined across studies (for example, adjustment, peer relationships, child health; Gottman et al., 1997; Lagacé-Séguin & Coplan, 2005), demonstrating different patterns of associations with emotion-coaching parenting. Second, different age settings and cultural contexts were explored across studies, generating diverge results in different developmental and social stages (e.g., Gottman et al., 1996, 1997; Lunkenheimer et al., 2007), as well as the specific characteristics of the school environment. Finally, emotion-coaching parenting can be more or less influential and significant given the educational environment in which the child is situated: whereas previous findings indicated significant associations between emotion-coaching parenting and children’s outcomes (e.g., Cunningham et al., 2009), fewer studies have found associations between this parenting style and older children’s outcomes. Kindergarten children, for example, are less expected to individually and independently cope with challenging situations compared to elementary school children, who spend more time on their own, both in class and during breaks. The different environment may play a role in the way in which emotional parenting is associated with children’s behavioral outcomes (Lagacé-Séguin & Coplan, 2005), as supported by our findings.

Interestingly, while no significant associations were found in our sample, this result is somewhat unexpected given prior theoretical and empirical work. Emotion-coaching parenting has been linked in several studies to enhanced emotion regulation, prosocial behavior, and academic outcomes in children (Gottman et al., 1997; Katz & Gottman, 1997; Katz & Windecker-Nelson, 2004; Shipman et al., 2007). It is therefore important to further examine this association across developmental stages and cultural contexts, in order to identify the conditions under which emotion-coaching parenting contributes to children’s adjustment during school transitions.

## 5. Limitations and Future Research

This study is a first attempt to capture the nature of the associations between emotion-focused parenting styles and adjustment to first grade. As such, it offers important insights and contributions to the literature. It also has several limitations that warrant consideration. First, a larger sample size would enhance the statistical power and potentially enable the detection of more subtle associations among the variables, if these exist. The current sample, while adequate for the initial exploration of these associations, may have limited our ability to identify significant associations, particularly for variables with smaller effect sizes. Second, the homogeneity of the respondents represents a notable limitation, as mothers constituted the vast majority of the participants in this study. Although the child gender distribution was balanced (approximately 50% boys and 50% girls), the inclusion of more fathers in future research would provide a more comprehensive understanding of parental influences on children’s adjustment. Third, the internal consistency reliability coefficients (Cronbach’s alpha) for some measurement scales in our study were not optimal. Future studies should aim to strengthen the psychometric properties of these measures, which might naturally occur with larger, more diverse samples. Another limitation of the study is its exclusive reliance on questionnaire-based assessment methods, which captured only parents’ perceptions of the studied variables. Incorporating multiple assessment methodologies—particularly observational measures of parent–child interactions and child adjustment—would significantly enhance the validity and richness of findings in this domain. An additional limitation concerns the exclusion of academic adjustment from the scope of measurement. Although this decision was theoretically grounded and aligned with the study’s focus on emotional and social aspects of the first grade transition, it may limit the generalizability of the findings. Including academic adjustment indicators in future studies would allow for a more comprehensive understanding of children’s overall school adjustment. The study also did not include teacher reports or other school-based data sources. While parental assessments provide valuable perspectives, incorporating information from teachers or classroom observations would yield a more comprehensive picture of children’s adjustment in the school context. Future research should adopt a multi-informant approach to better capture the dynamics of school adjustment.

Thus, while our findings contribute valuable insights in understanding the relationships between emotion-focused parenting styles and children’s adjustment to first grade, future research should address these limitations by utilizing larger and more diverse samples, enhancing the measurement reliability, incorporating multiple assessment methodologies, and including a broader range of adjustment outcomes—particularly academic indicators—to provide a more holistic and accurate representation of school adjustment.

## 6. Conclusions

The current study provides valuable insights into the role of emotion-focused parenting styles in children’s social and emotional adjustment to first grade. The findings highlight the importance of considering emotional aspects of parent–child relationships in understanding children’s developmental outcomes during this critical transition period, particularly, in terms of negating and minimizing the role of emotions in the experience of the child. The differential patterns of associations between various parenting styles and adjustment outcomes, as well as the moderating role of emotion regulation, suggest complex pathways through which parenting is associated with children’s development. These findings suggest promising theoretical paths for scholars to further pursue these associations, and they have important implications for interventions aimed at supporting children and families during the transition to formal schooling.

## Figures and Tables

**Table 1:** Descriptive statistics and bivariate correlations among the studied variables.

	M	SD	1	2	3	4	5	6
1.Dismissive Parenting	2.52	0.36	-					
2.Disapproving Parenting	2.39	0.50	0.42 **	-				
3. Laissez-Faire Parenting	3.09	0.44	0.11	0.000	-			
4. Emotion Coach Parenting	3.63	0.44	−0.03	−0.05	0.05	-		
5. Emotional Adjustment	3.63	0.53	−0.02	−0.21 **	−0.08	0.06	-	
6. Social Adjustment	4.08	0.44	−0.18 *	−0.26 **	−0.17 *	−0.01	0.48 **	-

N = 210, * *p* < 0.05, ** *p* < 0.01.

**Table 2:** Dismissing parenting style predicting social adjustment to first grade.

	Step 1		Step 2		Step 3		Step 4	
Variable	se	*b*	se	*b*	se	*b*	se	*b*
Intercept	0.21	4.69 ***	0.23	4.43 ***	0.26	4.15 ***	0.38	5.23 ***
Dismissing parenting	0.08	−0.24 **	0.082	−0.23 **	0.08	−0.22 **	0.08	−0.23 **
Child gender			0.06	0.15 *	0.06	0.15 *	0.06	0.16 **
Family income					0.03	0.06 *	0.03	0.04
Emotion regulation							0.11	−0.42 ***

N = 210, * *p* < 0.05, ** *p* < 0.01, *** *p* < 0.001.

**Table 3:** Disapproving parenting style predicting social adjustment to first grade.

	Step 1		Step 2		Step 3		Step 4	
Variable	se	*b*	se	*b*	se	*b*	se	*b*
Intercept	0.15	4.67 ***	0.17	4.43 ***	0.21	4.16 ***	0.33	4.94 ***
Disapproving parenting	0.06	−0.25 ***	0.06	−0.24 ***	0.06	−0.24 ***	0.06	−0.20 ***
Child gender			0.06	0.15 *	0.06	0.15 *	0.06	0.16 **
Family income					0.03	0.06 *	0.03	0.05
Emotion regulation							0.11	−0.34 **

N = 210, * *p* < 0.05, ** *p* < 0.01, *** *p* < 0.001.

**Table 4:** Disapproving parenting style predicting emotional adjustment to first grade.

	Step 1		Step 2		Step 3		Step 4	
Variable	se	*b*	se	*b*	se	*b*	se	*b*
Intercept	0.19	4.17 ***	0.22	3.91 ***	0.27	3.55 ***	0.37	5.99 ***
Disapproving parenting	0.08	−0.23 **	0.08	−0.22 **	0.08	−0.21 **	0.07	−0.11
Child gender			0.08	0.16 *	0.08	0.16 *	0.06	0.18 **
Family income					0.04	0.09 *	0.03	0.03
Emotion regulation							0.12	−1.06 ***

N = 210, * *p* < 0.05, ** *p* < 0.01, *** *p* < 0.001.

**Table 5:** Laissez-faire parenting style predicting social adjustment to first grade.

	Step 1		Step 2		Step 3	
Variable	se	*b*	se	*b*	se	*b*
Intercept	0.22	4.63 ***	0.24	4.38 ***	0.34	5.35 ***
Laissez-faire parenting	0.07	−0.18 *	0.07	−0.17 *	0.07	−0.16 *
Child gender			0.06	0.15 *	0.06	0.16 **
Emotion regulation					0.11	−0.43 ***

N = 210, * *p* < 0.05, ** *p* < 0.01, *** *p* < 0.001.

## Data Availability

Due to the sensitive nature of the data and the ethical approval granted by the University of Haifa’s Ethics Committee, the dataset is not publicly available. However, it may be shared upon reasonable request with qualified researchers, subject to ethical and legal review.
